# Swarm Learning And Clinical Data: Evaluating The Benefit of a New Node For The Whole Network

**DOI:** 10.1007/s41666-026-00255-7

**Published:** 2026-10-03

**Authors:** Matteo Mantovani, Simone Scaglia, Riccardo Scheda, Carlo Combi, Stefano Diciotti

**Affiliations:** 1 Department of Computer Science, University of Verona, Verona, Italy; 2 SuperComputing Applications and Innovation (SCAI), Cineca, Italy; 3 Department of Electrical, Electronic, and Information Engineering “Guglielmo Marconi”, University of Bologna, Bologna, Italy

**Keywords:** Data distribution, Distributed learning, EHR, KPI, Performance analysis, Swarm learning

## Abstract

Digital technologies in healthcare have significantly enhanced clinical decision-making with complex data analysis. However, single clinical centers often lack sufficient data volume or diversity for effective modeling. Distributed learning solutions help to overcome these limitations, and in particular, Swarm Learning (SL) offers a privacy-preserving solution by allowing a decentralized and synchronized model training across multiple centers without direct data sharing. Swarm Learning’s inherent focus on data privacy makes it well-suited for distributed healthcare analysis. However, no studies have quantified the impact of an external individual center that wants to join the swarm network in terms of performance and fairness. In this paper, we propose an approach for evaluating the benefits from the point of view of both a new center and the existing swarm network, based on the amount of data introduced by the new center. We also present seven different Key Performance Indicators (KPIs) to measure the impact of the new center under different aspects related to the performance variation, model stability, fairness, and quality of the newly introduced data. We apply these indicators using real Intensive Care Unit (ICU) data from the *MIMIC-III* and *MIMIC-IV* datasets, and we analyze the impact of the new center based on different data distribution scenarios and different numbers of centers in the network.

## Introduction

The clinical field has increasingly embraced digital technologies to improve patient care and, more broadly, to transform the management and experience of healthcare. Today, numerous hospitals and healthcare organizations utilize digital solutions, and some clinical datasets are accessible to researchers and healthcare professionals. Analyzing these datasets plays a crucial role in decision-making processes, such as resource allocation, patient care strategies, disease progression forecasting, and the development of treatment protocols [1].

Nevertheless, there could be circumstances in which the data collected by a single center is not abundant or adequate enough to perform proper learning (e.g., rare disease analysis). In such scenarios, conducting analyses across multiple servers can be advantageous. However, many clinical centers are unable or unwilling to share data with others due to concerns such as privacy. To address this challenge, privacy-preserving distributed algorithms have been developed, including Secure Multiparty Computation (SMPC) [2], homomorphic encryption [3], and more recently, federated learning [4]. These approaches have attracted particular attention in healthcare, where they can support multi-institutional model development while limiting the need for direct patient-data sharing [5, 6].

Federated learning enables model training across multiple centers without data sharing. Instead of sharing raw data, nodes train local models on their local datasets and periodically exchange parameters, such as the weights of a deep neural network, to collaboratively build a global model. However, federated learning typically relies on a central server for parameter sharing and synchronization, which can reduce fault tolerance [7].

**Fig. 1:**
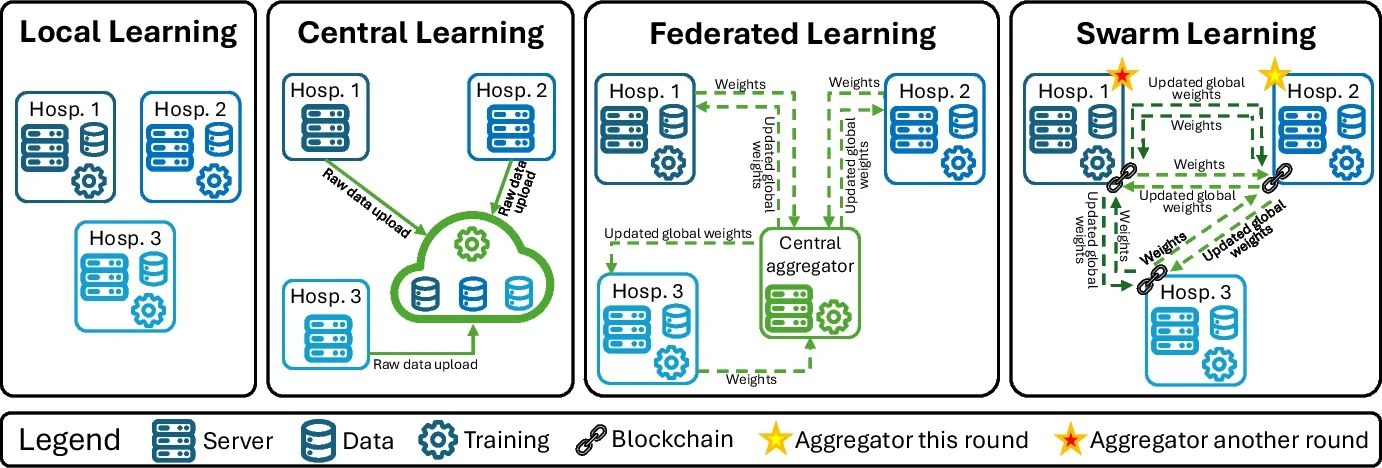
Different learning architectures

Swarm Learning (SL) is a technique introduced in 2021 by Warnat-Herresthal et al. [7] whose goal was to address the limitation of centralized control through a distributed and synchronized training without requiring a permanent central server. SL is based on the concept of nodes, which are components that perform specific functions and collaborate with others within the network. Each node processes data independently and contributes to the collective learning task, which benefits from the diversity of data samples of individual nodes. The approach enhances privacy by ensuring that data remains localized on each node. The central server is not needed, as parameter sharing and synchronization are managed by an epoch leader, i.e., a node randomly selected at each synchronization step. Figure 1 shows the different learning architectures, highlighting the enhanced focus for data privacy with federated and swarm learning, and the decentralized aspects of the SL.

The original paper introducing SL [7] explored various training configurations, including scenarios with uneven data distribution among nodes and a limited examination of heterogeneous disease data distributions. However, the study did not compare SL-trained models to centralized server models, where training occurs on the entire dataset in a single location. Additionally, the authors employed a simple averaging method for parameter merging without investigating alternative approaches involving weighted contributions from nodes.

Recent studies have further examined SL performance and its applications in healthcare [8, 9], but without a systematic analysis of SL performance when data availability varies significantly across nodes. In [10], the authors performed such an analysis, considering the shift in the performance of a reference node based on its data distribution with respect to the other nodes in the network. However, this approach considered only nodes already belonging to the swarm network, and there is no analysis on the benefits for a *new* node to join the swarm learning network.

For example, let us consider a swarm network composed of four different clinical centers, and a new node– unknown to the network– is considering joining it. What is the expected advantage, in terms of learning performance, for the node to enter the network, compared to locally training a model? And is there also a benefit for the swarm network itself? Does this improvement change based on the distribution of the data between this new node and the others? Is the considered dataset relevant to this analysis, or does SL behave with the same pattern in every dataset?

In this paper, we aim to address these questions by systematically evaluating the performance of SL under various data configurations across participating nodes. Our investigation focuses on analyzing how a swarm network adapts and performs when a newly introduced node contributes unique data that has not previously been utilized in the learning process of other nodes. In this setting, the expected performance improvement associated with additional homogeneous data is not the main focus of the study; rather, it represents the controlled condition under which the contribution of the new node can be isolated and interpreted. Through this analysis, we want to propose a framework for checking the adaptability and robustness of the SL framework in handling different data distributions with multiple datasets.

To achieve this, we define and apply a set of Key Performance Indicators (KPIs) that provide a detailed analytical framework to measure the impact of introducing a new node, as well as the overall performance of the swarm network under different data scenarios. These KPIs are designed to quantify the new node’s incremental gains and assess the collective benefits to the swarm network as a whole. Finally, we validate our proposed methodology by applying it to the Intensive Care Unit (ICU) data from the MIMIC-III and MIMIC-IV datasets, using the swarm learning free tool provided by HP Enterprise named *HPE Swarm Learning Community*. Through this evaluation, we analyze the outcomes of our analysis and discuss the results, focusing on the scalability and adaptability of swarm learning in real-world clinical contexts. This study should be interpreted as a controlled baseline for evaluating the benefit of adding a new node to an existing swarm network. Therefore, we deliberately avoid non-IID data in order to isolate the impact of the new node’s data volume from confounding effects due to feature or label heterogeneity.

## Background and Related Work

SL was initially introduced in 2021 by Warnat-Herresthal et al. [7] as a variation of federated learning that removes the need for a central coordinator (server) by adding a decentralized coordination that relies on a blockchain-based peer-to-peer network. That study explored the potential of using SL to develop disease classifiers using distributed data, focusing on four heterogeneous diseases: tuberculosis, leukemia, COVID-19, and lung pathologies. Such classifiers are usually chosen among well-known machine learning/deep learning techniques [11, 12]. Dataset sizes varied significantly, from 899 samples of a whole-blood-derived transcriptome dataset to 47,000 X-ray images. Their results demonstrated that, under their specific configuration, the SL model almost consistently outperformed models trained locally at each node. However, they did not compare the performance of SL-trained models with that of a centrally trained model, where the entire dataset is processed on a single server. Also, investigating uniform and non-uniform data distribution scenarios across nodes was very limited.

Following this initial work, other research groups have explored SL, particularly in clinical applications. For example, Saldanha et al. [8] trained deep learning models using SL on multicentric datasets comprising gigapixel histopathology images from over 5,000 patients, comparing the performance of SL models against models trained on a centralized dataset. Similarly, in [9], the authors collected digital whole-slide images (WSIs) of tissue samples from four cohorts of gastric cancer patients across different countries, resulting in over 2,000 samples. Three cohorts were used for training and one for testing.

In all these contributions, the model’s performance trained on centralized data often matched or exceeded that of SL models, but only in [10] did the authors perform a systematic analysis of SL performance under different data distribution scenarios. In particular, they analyzed a reference node already included in the swarm network, assigning different data portions to it while redistributing the remaining data among the other nodes. Thus, the total amount of data available to the swarm remained fixed across scenarios.

The present work addresses a different, although related, setting. Here, the baseline swarm is trained without access to the data partition assigned to the candidate new node, whereas the augmented swarm includes both the new node and its data. Therefore, the focus is not on redistributing a fixed dataset among nodes already present in the swarm, but on quantifying the impact of adding a new node and its data to an existing SL configuration. Moreover, to this end, we introduce a set of KPIs designed to characterize this contribution from multiple perspectives, including the impact on the new node, the overall swarm, and the pre-existing nodes.

### MIMIC-III

Medical Information Mart for Intensive Care III (MIMIC III) [1] is a publicly-available relational database, and it contains de-identified health-related information from more than 60 000 admissions for 46 000 unique patients admitted to critical care units at the Beth Israel Deaconess Medical Center (BIDMC) in Boston, Massachusetts, between 2001 and 2012. The database includes different kinds of information to compose a complete patient medical record. MIMIC-III includes vital sign measurements (automatically taken or manually reported), demographics, laboratory test results, prescriptions, procedures, diagnosis, caregiver notes, and many other valuable information to represent a complete description of a patient’s stay accurately. Data access is governed by the PhysioNet credentialing process, ensuring compliance with ethical and legal standards to protect patient privacy while promoting open scientific inquiry.

### MIMIC-IV

The Medical Information Mart for Intensive Care IV (MIMIC-IV) [13] database is a publicly available, large-scale dataset containing de-identified health-related data from almost 300 000 patients, over 430 000 admissions, and 73 000 ICU stays. MIMIC-IV builds upon its predecessor, MIMIC-III, with updated and expanded records. The data spans admissions between 2008 and 2019, offering a comprehensive view of critical care practices over more than a decade. MIMIC-IV includes data on patient demographics, vital signs, laboratory results, medications, procedures, and clinical notes. Its modular design organizes data into distinct tables for ease of navigation and integration with analytical tools. A key feature of MIMIC-IV is its temporal granularity, which captures time-stamped observations and interventions, enabling dynamic analyses of patient trajectories.

### HPE Swarm Learning Software

Hewlett-Packard Enterprise (HPE) developed a freely available SL software, named HPE Swarm Learning Community Edition^1^, that allows training collaborative models between different servers without sharing the training data. This is achieved by individual nodes sharing model weights derived from training the model on the local data of each node.

The SL process begins with enrollment on the swarm smart contract by each node. Next, nodes proceed to train a local copy of the model iteratively over multiple epochs, for a fixed number of iterations. After reaching the number of iterations, each node exports its model parameters and sends them to the epoch leader, which is chosen randomly at each synchronization step between all the nodes. The epoch leader merges all the models’ parameters from each node and sends the merged parameters to the other peers. Then, each node trains the model until the fixed iteration number is reached again. A final swarm model is obtained at the end of all the synchronization steps, and each node receives the swarm merged model.

The merging of weights is carried out using a predefined algorithm. Although the algorithm is not customizable, the official HPE package offers three options: weighted average, coordinate median, and geometric median.

The maximum number of nodes allowed in the HPE Swarm Learning Community edition is 5. However, there is complete flexibility in the location of these nodes. Indeed, it is possible to have a single node for each connected server, multiple nodes running on a single server, or a combination of both scenarios.

## Methodology

Thanks to its decentralized nature and emphasis on data privacy, the SL framework is well-suited for distributed healthcare analysis across multiple centers. Rather than focusing on SL’s security aspects, this study aims to evaluate its learning performance across various scenarios. In this section, we want to systematically analyze the effects of a single –*new*– node when it joins the swarm network, based on different data distributions between this node and all the others that were already part of the network. In particular, we want to analyze the impact of this new node on the swarm network, as well as the influence of this node on itself and the other nodes in terms of learning performance when different data distribution scenarios are considered. This analysis simulates different real-world situations in which the amount of data between centers differs. To do so, we considered this new node as a *reference* node with a given percentage of the training set, uniformly distributing the remaining part of it in the other nodes. Learning performance is evaluated by varying the percentage of the training set assigned to the new node, while evenly distributing the remaining portion across the other nodes. To ensure consistency in class representation, stratified sampling is used to maintain the target class distribution. This ensures that both target classes, including the less frequent sepsis class, remain represented across the different data splits according to their original proportions. However, several key factors may influence performance or must be considered to fully understand the causes of performance variation.

In this section, we propose several indicators to analyze the learning process’s performance and fairness when a new node joins the swarm network. The idea of using multiple indicators is based on the fact that we could analyze the variation in performance/fairness from different perspectives.

First of all, let us introduce some parameters:

- *N* represents the current number of nodes within the swarm network (*N≥ 2*);
- *N'* is used to identify a swarm network with *N* nodes plus the new node that joined (i.e., *N' = N + 1*)
- $p_i \in [0,1]$ describes the performance of the *i*-th node, trained on its local data. In particular:
  - $p_{new} \in [0,1]$ refers to the performance of the *new* node;
  - $p_{others} \in [0,1]$ indicates the *average* performance of all the other nodes. That is:
    1 $$\begin{aligned} p_{others} = \sum _{i=1}^{N} \frac{p_i}{N} = \sum _{i=1,i\ne {new}}^{N'} \frac{p_i}{N'-1} \end{aligned}$$
- $sp_N$, $sp_{N'}$ indicate the swarm model performance (*sp*) with *N*, *N'* nodes ($sp_N, sp_{N'}\in [0,1]$), respectively;
- $sp_N^{i}$, $sp_{N'}^i$ indicate the performance of the *i*-th node on the swarm model with *N* and *N'* nodes, respectively ($sp_N^{i}, sp_{N'}^i \in [0,1]$); In particular:
  - $sp_{N'}^{new} \in [0,1]$ refers to the performance of the *new* node;
  - $sp_N^{others}$ indicates the *average* performance of all the other nodes on the swarm model without the new node ($sp_N^{others}\in [0,1]$);
  - $sp_{N'}^{others}$ indicates the *average* performance of all the other nodes on the swarm model with the new node ($sp_{N'}^{others}\in [0,1]$).
- $w_i$ specifies the weight of the *i*-th node, $w_i \in [0,1]$, which, in this scenario, corresponds to the percentage of the training set within that node.

As mentioned before, the *new* node is evaluated with different data distributions, and all the other nodes equally receive the remaining training subset. Here, the percentage of data received corresponds to the weight of the node. Thus, while the weight of the *new* node ($w_{new}$) is independent, the weight of all the other nodes ($w_{others}$) is complementary and equal across nodes:

2 $$\begin{aligned} w_{others} = w_{o_1} = w_{o_2} = \dots = w_{o_{N}} \end{aligned}$$

And the sum of all the weights is:

3 $$\begin{aligned} w_{new} + N w_{others} = 1 \end{aligned}$$

### Swarm Gain

The swarm gain ($S_g$) is a very essential indicator, as it compares the performance of the swarm learning before and after a *new* node joins the network. This is defined as:

4 $$\begin{aligned} S_g = \frac{sp_{N'}}{sp_N} \end{aligned}$$

The swarm gain should return us an overall first indication regarding the impact of an additional node in the network. In fact, a swarm gain greater than 1 indicates that the swarm network improves its performance with the new node, while a value lower than 1 indicates the opposite.

### Local Gain

From the perspective of this new node, it could be interesting to measure the gain/loss in performance from its locally trained data ($p_{new}$) to the swarm learning network $sp_{N'}$. Here, we can observe whether this node takes advantage of joining the swarm network or vice versa. The local gain ($L_g$) is defined as:

5 $$\begin{aligned} L_g = \frac{p_{new}}{sp_{N'}} \end{aligned}$$

A local gain greater than 1 indicates that the local model is performing better than the swarm learning, so it may seem there is no real advantage for this node to join the network. On the contrary, when $L_g < 1$, the new node benefits from the swarm network as it improves the performance over its local one. However, let us remember that this score is valid only for the considered dataset, and the situation could change with different data. Also, if the local gain is lower than 1 on the new node, we should consider circumstances where all the nodes represent different clinical centers in the same network. Here, this new node, which is part of the same clinical network, could help all the other nodes to improve their performance.

### Relative Local Gain

Complementarily, we could retrieve the effect of the new node on the other nodes that were already part of the swarm network. For this purpose, we could introduce the relative local gain ($RL_g$), where the term “local” refers to the pre-existing nodes in the swarm network rather than to locally trained models. It is defined as:

6 $$\begin{aligned} RL_g = \frac{sp_{N'}^{others}-sp_N^{others}}{sp_N^{others}} \end{aligned}$$

7 $$\begin{aligned} RL_g = \frac{sp_{N'}^{others}-sp_N^{others}}{sp_N^{others}} \end{aligned}$$

Here, we can compare the relative performance of the other nodes with respect to their swarm performances before and after the new node joins. If $RL_g > 0$, the other nodes perform better in the swarm network after the new node joins. That is, the new node contributed positively to the learning process of the swarm network, and the other nodes benefit even more from being in it. On the contrary, a value lower than 0 suggests that the new node is reducing the benefits for the other nodes to be in the swarm network.

However, if the swarm performance is calculated through the average performance of the single nodes ($sp^i_N$), the relative local gain could be related to the swarm gain indicator ($S_g$) if we assume that the swarm learning algorithm runs for enough epochs. Indeed, in this context, the performances of all nodes, including the newly joined node, are expected to converge towards similar values. Thus, it could also be used to verify if the number of epochs adopted is enough in the training phase. First of all, with the average performance, we obtain that:

8 $$\begin{aligned} sp_N = sp_N^{others} \end{aligned}$$

Then, we also know that with a proper number of epochs, all the nodes converge on similar performance values. Consequently, if $sp_{N'}^{new} \approx sp_{N'}^{others}$, the average swarm performance of the new network could be approximated to:

9 $$\begin{aligned} sp_{N'} = sp_{N'}^{others} \end{aligned}$$

And the whole $RL_g$ formula could be rewritten as:

10 $$\begin{aligned} RL_g = \frac{sp_{N'}-sp_N}{sp_N} = \frac{sp_{N'}}{sp_N} - \frac{sp_N}{sp_N} = \frac{sp_{N'}}{sp_N} - 1 = S_g - 1 \end{aligned}$$

That is, if the swarm performance of the new node converges to the one obtained by the other nodes, the relative local gain and the swarm gain indicators are directly related. However, both metrics are intended to show different aspects of the swarm network and, even though they can partially overlap in the homogeneous converged setting, they remain conceptually distinct KPIs.

### Model Stability

To assess the robustness of the swarm learning process, we define the Model Stability Indicator (MSI), which quantifies the variation in model performance before and after the inclusion of the new node. This metric evaluates whether the integration of this new node introduces instability or maintains consistency in the learned model. MSI is computed as the relative standard deviation of the swarm model’s performance, comparing its variance before and after the new node joins. Formally:

11 $$\begin{aligned} MSI = \frac{\sigma _{sp_{N'}}}{sp_{N'}} - \frac{\sigma _{sp_N}}{sp_N}. \end{aligned}$$

Where $\sigma _{sp_{N'}}$ and $\sigma _{sp_{N}}$ respectively denote the standard deviation of all the swarm nodes’ model performance before and after the new node joins, and $sp_{N'}$ and $sp_{N}$ are the corresponding mean performances, respectively. The standard deviations are defined as:

12 $$\begin{aligned} \sigma _{sp_N} = \sqrt{\frac{1}{N}\sum _{i=1}^{N} \left( sp_N^i\right) ^2 - \left( sp_N\right) ^2} \end{aligned}$$

13 $$\begin{aligned} \sigma _{sp_{N'}} = \sqrt{\frac{1}{N+1}\left[ \sum _{i=1, i\ne {new}}^{N'} \left( sp_{N'}^i\right) ^2 + \left( sp_{N'}^{new}\right) ^2\right] - \left( sp_{N'}\right) ^2} \end{aligned}$$

This indicator is particularly useful for evaluating the behavior of the swarm network when it is afflicted by dynamic changes in node composition. A positive MSI indicates increased instability in model performance, potentially suggesting divergence due to the new node. On the contrary, a negative MSI suggests improved consistency in swarm learning. An MSI close to zero implies minimal impact on model stability.

### Jain’s Index

In the SL environment, it is crucial not to evaluate only the overall performance, but also to assess the fairness of the nodes’ contributions. This is essential to ensure that the global model is not heavily influenced by a single node. In fact, even though the global model is obtained by aggregating local updates (i.e., sharing model parameters) from all the nodes, if one node achieves a significantly higher performance on its local dataset, its updates will have a stronger influence during aggregation. As a result, the global model’s parameters could become more reflective of that node’s data distribution. Therefore, the final-global model is uniformly distributed to all nodes, but it remains biased toward the predominant node, performing well on data similar to that node while potentially underperforming on other nodes. This imbalance undermines the overall generalizability and fairness of the model, and, as a result, it could negate the collaborative advantage of SL. To quantify fairness, we employ Jain’s index [14], defined as:

14 $$\begin{aligned} J = \frac{\left( \sum _{i=1}^{N'} sp_{N'}^i\right) ^2}{N' \sum _{i=1}^{N'} (sp_{N'}^i)^2} \end{aligned}$$

A Jain’s index close to 1 indicates that the performance contributions are evenly distributed among the nodes, ensuring that the model is not excessively biased toward any single node. On the other hand, a lower Jain’s index reveals an imbalance in contributions, suggesting that a dominant node may bias the aggregated model. It could be interesting to examine Jain’s index together with other indicators, such as the swarm gain ($S_g$) and the Data Utility Index (defined in the next Section), in order to assess not only the fairness in the global model, but also the diversity of the data introduced and the variation in terms of performance altogether.

### Data Utility Index

All the indicators illustrated before directly analyze the performance and the fairness of the swarm learning. However, it is also important to investigate the data analyzed to quantify the impact of a new node joining the swarm network, and, for this purpose, we propose the Data Utility Index (DUI). In this paper, we analyze different data distribution scenarios, and we use stratification to preserve the target class distribution. However, it could be common for each node to contain data with all possible labels for a given feature. DUI is designed to measure the novelty of data introduced by a new node in a distributed learning system. Unlike simple count-based measures, DUI leverages the full probability distribution of categorical features to assess how different the new node’s label distributions are from those of the existing nodes. This approach is particularly meaningful when each node is expected to contain all possible labels (i.e., values) for a given feature; under these conditions, simply counting the labels is insufficient, and a comparison of their full probability distributions becomes essential.

Considering the set *F* of features, for each feature *f ∈ F* with label set $L_f$, we compute the probability distributions

15 $$\begin{aligned} P_{f,\text {new}}(l)=\frac{\text {Count of label } l \text { in new node data}}{\text {Total new node observations}},\quad l\in L_f \end{aligned}$$

and

16 $$\begin{aligned} P_{f,\text {others}}(l)=\frac{\text {Count of label } l \text { in other nodes data}}{\text {Total other observations}},\quad l\in L_f \end{aligned}$$

We then define the midpoint distribution as

17 $$\begin{aligned} M_f(l)=\frac{1}{2}\Bigl (P_{f,\text {new}}(l)+P_{f,\text {others}}(l)\Bigr ),\quad l\in L_f \end{aligned}$$

The discrepancy between $P_{f,\text {new}}$ and $P_{f,\text {others}}$ is measured using the Jensen-Shannon divergence [15, 16]:

18 $$\begin{aligned} \begin{aligned} D_{\textit{JS}}(P_{f,\text {new}}, P_{f,\text {others}}) = \\ \frac{1}{2}\sum _{l \in L_f} P_{f,\text {new}}(l)\ln \frac{P_{f,\text {new}}(l)}{M_f(l)} \\+ \frac{1}{2}\sum _{l \in L_f} P_{f,\text {others}}(l)\ln \frac{P_{f,\text {others}}(l)}{M_f(l)} \end{aligned} \end{aligned}$$

The overall DUI for the new node is then defined as the average divergence over all features in *F*:

19 $$\begin{aligned} \text {DUI}_{\text {new}}=\frac{1}{|F|}\sum _{f=1}^{F} D_{\textit{JS}}(P_{f,\text {new}}, P_{f,\text {others}}) \end{aligned}$$

Since the Jensen-Shannon divergence is bounded between 0 and 1, a DUI score of 0 indicates identical distributions, while higher values suggest that the new node’s contributing data are substantially different from what is already present. This difference can be interpreted in two ways: if the new data are more diverse, they could increase the overall representativeness and robustness of the model; alternatively, if the divergence is too pronounced, it could indicate potential inconsistencies or mismatches with the already existing data. Thus, the DUI serves as an important diagnostic tool to analyze how well the new node’s data aligns with or deviates from the aggregated patterns of the existing nodes.

### Entropy

In addition to the DUI, we aim to further investigate the impact of the new node, particularly whether it introduces novel information, by computing the label entropy (*H*). Higher entropy denotes a wider variety in the new set of labels, thus suggesting that the new node contributes a range of previously unseen information. A lower entropy implies the prevalence of some labels that may limit the contribution to model diversity.

In order to evaluate the impact of the new node’s entropy on swarm learning, we have to analyze the entropy across the entire swarm network before and after this new node joins it. We first compute the overall label entropy of the swarm before the new node joins, denoted as $H_{sn\_N}$, and then compute the overall label entropy after the new node joins the network, denoted as $H_{sn\_N'}$.

For our entropy evaluation, we rely on Shannon entropy [17]. That is, for a given dataset, the label entropy is defined as:

20 $$\begin{aligned} H = -\sum _{i=1}^{K} \text {prob}_i \ln \left( \text {prob}_i\right) , \end{aligned}$$

Where:

- *K* is the total number of distinct labels,
- $\text {prob}_i$ is the probability of label *i*, computed as
  $$ \text {prob}_i = \frac{N_i}{N_{\text {total}}} $$
  with $N_i$ being the number of occurrences of label *i* and $N_{\text {total}}$ the total number of samples.

The maximum possible entropy, which occurs when all labels are equally likely, is given by

21 $$\begin{aligned} H_{\text {max}} = \ln (K) \end{aligned}$$

Normalizing the entropy yields

22 $$\begin{aligned} H_{\text {norm}} = \frac{H}{\ln (K)} \end{aligned}$$

This normalization bounds $H_{\text {norm}}$ between 0 and 1.

We then define the change in normalized entropy due to the addition of the new node as

23 $$\begin{aligned} \Delta H_{\text {norm}} = H_{norm, sn\_N'} - H_{norm, sn\_N} \end{aligned}$$

By correlating $\Delta H_{norm}$ with the swarm gain ($S_g$), we can assess whether an increase in label diversity leads to improved performance (i.e., $S_g > 1$) or if the additional diversity introduces noise that reduces the learning performance. A positive $\Delta H_{\text {norm}}$ accompanied by an $S_g > 1$ suggests that the novel information from the new node enriches the network. In contrast, a negative correlation may indicate that excessive diversity penalizes the swarm learning process.

## Experimental Evaluation

In this section, we present our experimental evaluation of all the indicators described in Section 3 using real clinical data. Specifically, we assess how performance and fairness vary, both from the perspective of individual nodes and the overall swarm network, when a *new* node joins an existing swarm, under different configurations and data distributions.

Moreover, our goal is to minimize the influence of “external” factors that could interfere with the evaluation of the impact of this new node besides the number of nodes and data distribution. Thus, we introduced some constraints. First of all, all the nodes in the network (the new one and the others) share the same distribution of the classes, as the dataset is split through stratification. We then deliberately selected the homogeneous data available in the *MIMIC-III* and *MIMIC-IV* datasets to ensure that heterogeneity would not interfere with our analysis. The resulting datasets, obtained through the process described in Subsection 4.2 were subsequently split into 70% for training and 30% for testing. The test sets are used to evaluate the performance metrics across all nodes in the swarm network.

The data distribution for the *new* node—used as the reference node—ranges from 2.5% to 90% of the original training set. The remaining portion of the dataset is evenly divided among the other nodes in the network, resulting in non-overlapping subsets obtained through stratified sampling. Table 1 details all the different data distributions and the corresponding weights used in our evaluation for a 5-node configuration. The same logic applies when using a different number of nodes. When training is performed prior to the *new* node joining the network, both its data and associated weight are entirely excluded from the process. Each node independently trains its model, and parameters are periodically merged with those of the other nodes. After swarm learning is completed, each node evaluates its model on the test set. Results are averaged over five independent training runs, each using a different random seed to shuffle the data.

Finally, it is important to note that the HPE Swarm Learning package represents node weights exclusively as integer values in the range [1–100] (see the official documentation^2^). Due to this limitation, we approximate the weights derived from decimal data distribution percentages to their nearest integer values, as shown in Table 1.

To summarize, we performed five training runs involving only the nodes initially present in the swarm network, where each node had the same amount of data and the same proportion of the target class. In these runs, the nodes used a subset of the training set that excluded the final, complementary portion later assigned to the *new* node upon its introduction into the network. We then repeated the same five training runs, this time including the *new* node, in order to assess its overall impact. This entire procedure was repeated across different data distribution scenarios between the *new* node and the existing nodes. The nodes already in the network kept the exact same data portion of the previous runs, so any difference is attributable to the *new* node itself.

**Table 1:** Percentage of data assigned to each node and corresponding integer weights across different data distribution configurations involving five nodes. Weights are expressed as integers, in accordance with the HPE Swarm Learning tool (v2.2), which supports only integer values for the weighted averaging process

Fold size (%)					Weights				
New Node	Other nodes				New Node	Other nodes			
2.5	24.375	24.375	24.375	24.375	2	24	24	24	24
5	23.75	23.75	23.75	23.75	5	23	23	23	23
10	22.5	22.5	22.5	22.5	10	22	22	22	22
20	20	20	20	20	20	20	20	20	20
30	17.5	17.5	17.5	17.5	30	17	17	17	17
40	15	15	15	15	40	15	15	15	15
50	12.5	12.5	12.5	12.5	50	12	12	12	12
60	10	10	10	10	60	10	10	10	10
70	7.5	7.5	7.5	7.5	70	7	7	7	7
80	5	5	5	5	80	5	5	5	5
90	2.5	2.5	2.5	2.5	90	2	2	2	2

### System configuration and software

As discussed in the previous sections, we want to analyze the impact of a *new* node joining the swarm network in terms of learning performance and fairness, and this analysis is performed with different data distribution scenarios between this new node and the others. Consequently, to reflect the percentage of distribution of the dataset, we adopted the *weighted average* as a merging algorithm, thus giving a weight value to each node that corresponds to the percentage of the training set within it.

The community edition of the HPE Swarm Learning software only allows up to 5 nodes, so we performed our experimental evaluation in three different scenarios: a new node joining a swarm network of 2, 3, and 4 existing nodes, where all of them simulate different clinical centers. However, it is important to highlight that the performance and fairness indicators adopted in this paper specifically focus on the number of nodes and their data distribution. Consequently, the obtained results are unaffected by the physical location of the nodes. For this reason, and to speed up the synchronization process, we run all the nodes within two servers located on the same rack. They are two Linux workstations equipped with a 16-core AMD Opteron(tm) Processor 4386 CPU and 16 GB RAM for each one. One runs on Ubuntu 20.04 LTS, while the other on Ubuntu 22.04 LTS. They both use the HPE Swarm Learning Community edition v. 2.2.0. With this hardware configuration, the computation time is about 29.5 hours for the entire evaluation with all the different data distributions on *MIMIC-III*, and 45 hours on *MIMIC-IV*.

This version of the HP Enterprise Swarm Learning package, which is the latest, is only compatible with deep learning models. So, we decided to create an Artificial Neural Network (ANN) with one input layer, three hidden layers, and one output layer. The three hidden layers are densely connected, each with 16 nodes and utilizing a rectified linear unit (ReLU) activation function [18]. The output layer is also densely connected and contains a single node with a sigmoid activation function.

The model configuration for training involves the use of the Adam optimization algorithm [19] to effectively minimize the binary cross-entropy loss between the true and predicted labels. This model is consistently applied across two different scenarios, which are the training on individual nodes using their local datasets, and within the swarm learning framework. Then, to ensure a robust learning process, the model undergoes training for a total of 25 epochs, using a batch size of 64 and a learning rate of 0.0001. We adopted a fixed training budget of 25 epochs for all experiments to ensure comparable training conditions across local, swarm, and centralized-data configurations. In the considered HPE Swarm Learning setting, validation-based early stopping is not directly applicable as a global swarm-level criterion, and local early stopping would monitor node-specific validation metrics and would not guarantee the selection of the best synchronized swarm model. Moreover, if different nodes stopped training at different epochs, they would contribute to subsequent synchronization steps after a different number of local updates, reducing the comparability of the training process across nodes. The area under the receiver operating characteristic curve (AUROC) was chosen as the primary metric for evaluating the performance of the model, as the task is a binary classification.

### Datasets ETL

In this subsection, we describe the process of Extract, Transform, Load (ETL) performed on *MIMIC-III* 1.4 and *MIMIC-IV* 2.2 to obtain a dataset ready to be used for the training and test of the swarm learning performances. We decided to adopt the MIMIC datasets as they are freely available and they contain a relevant number of ICU patients with over a decade of timestamped data for each dataset, but from *MIMIC-III* we use only the data recorded through the MetaVision system, used also in *MIMIC-IV* [1, 13].

Briefly, for this analysis, we focused on patients diagnosed with sepsis during their stay, and this represents our class of interest (i.e., *true* if the patient has been diagnosed with any kind of sepsis, *false* otherwise). Then, we selected a set of drugs that are known to be heavily or weakly related to sepsis treatments [20, 21] along with several vital signs.

In detail, we started by extracting the patients by applying an explicit age filter (admission time - date of birth (*MIMIC-III*) or anchor_age (*MIMIC-IV*) between 18 and 89) to exclude pediatric and neonatal cases, focusing solely on adult patients whose clinical trajectories are distinct from younger populations. We also excluded some very elderly patients, as in the MIMIC datasets all the patients over 90 years old are grouped together for their anonymization purposes, so it is not possible to obtain the real age. It is worth mentioning that we filtered by the patients’ age during their admissions; that is, if a patient is admitted multiple times, we consider only those within the age range considered, and each admission is treated separately from the others. In this setting, each row of the final table corresponds to a single admission, while each column contains an element of the patient’s medical history selected as a feature for the analysis.

The vital signs were extracted from the chartevents table, focusing on the fundamental parameters that are indicators of the patients’ status, especially when they are diagnosed with sepsis. The vital signs considered were heart rate (id=220045), respiratory rate (id=220210), oxygen saturation (id=220277), body temperature (id=223761 and 223762 to include both Celsius and Fahrenheit), and white blood cell count (id=220546), which may be critical in monitoring the reaction of the immune system during sepsis. Systolic and diastolic blood pressure was also retrieved both as arterial (id=220050 and 220051) and non-invasive (id=220179 and 220180), as separate vital signs. Following the thresholds indicated in the guidelines, all these values were then discretized as “low”, “normal”, and “high”, as described in Table 2. Also, to ensure data quality and reliability, implausible or physiologically impossible values (e.g., extreme outliers) were excluded, reflecting our goal to obtain clean data for a robust analysis. For all the vital signs described, we created three different features containing the label for the minimum, maximum, and average measurements recorded during the admission. To ensure a sufficient number of measurements—typically recorded at least once per hour—we included only admissions containing at least one ICU stay of at least one day. This choice allowed us to exclude short-term admissions and focus on cases more likely to involve significant interventions, critical care requirements, and more comprehensive clinical data.

**Table 2:** Thresholds used to discretize the considered vital signs into “low” and “high” categories. Values falling between these thresholds are labeled as “normal”, while those outside the defined ranges are considered likely to be incorrectly reported, as they fall outside physiologically plausible limits. An exception is made for SpO$_2$, which is expressed as a percentage: values between 96 and 100 are labeled as “normal”, and no “high” category is defined for this variable

	Heart Rate (bpm)	Diastolic BP (mmHg)	Systolic BP (mmHg)	White Blood Cells (K/*μ *L)	Body Temp. ($^{\circ }$C)	Resp. Rate (insp/min)	SpO$_2$ (%)
Low	10 *≤ * val *≤ * 60	10 *≤ * val *≤ * 60	50 *≤ * val *≤ * 90	0 *≤ * val *≤ * 4.0	15 *≤ * val *≤ * 36	4 *≤ * val *≤ * 12	40 *≤ * val *≤ * 95
High	100 *≤ * val *≤ * 300	90 *≤ * val *≤ * 300	140 *≤ * val *≤ * 400	11.0 *≤ * val *≤ * 100	38.3 *≤ * val *≤ * 50	20 *≤ * val *≤ * 120	–

We then focused on the drug administration by selecting a set of drugs usually recommended to treat sepsis by the guidelines [20, 21] along with weakly sepsis-related medications. The data is sourced from the inputevents_mv table for *MIMIC-III*, and from inputevents and emar tables for *MIMIC-IV*. The drugs considered include vasopressors such as norepinephrine, epinephrine, phenylephrine, vasopressin, and dopamine, which are used to manage hypotension and septic shock; antibiotics like vancomycin and piperacillin/tazobactam, which are commonly administered for broad-spectrum antimicrobial coverage in sepsis; potassium chloride, which is used for electrolyte correction; omeprazole and pantoprazole as proton pump inhibitors; metoprolol as a beta-blocker. Additionally, ciprofloxacin and acyclovir were included due to their occasional but non-standard use. Let us note that while some of these medications are essential in sepsis treatment, others—such as metoprolol, potassium chloride, phenylephrine, omeprazole, and pantoprazole have more general applications beyond sepsis management. In this ETL process, each drug mentioned is a feature we consider, and we mark it as (*true*; *false*) if the patient had it during the stay. However, we consider only the stays with at least 3 drug administrations to focus, again, on hospitalizations likely related to relevant interventions.

Lastly, sepsis cases are extracted from table diagnoses_icd, and, in the end, we collected 41 different features. The final table with data from *MIMIC-III* counts 17 569 patients’ admissions with 2408 cases of sepsis (13.7%), while the table with *MIMIC-IV* data counts 51 100 admissions with 8556 cases of sepsis (16.7%).

For the experimental evaluation on *MIMIC-III*, we had 12 298 samples in the training set and 5271 samples for the test set, keeping the same proportions of patients with sepsis in the two sets. With *MIMIC-IV*, we had 35 770 and 15 330 samples for the training and test sets, respectively.

### Results and Discussion

In the following, we will illustrate and discuss the results obtained through the analysis of a swarm network with up to 5 nodes, when a *new* node joined the network. First of all, we need to calculate our reference performance, that is, the average AUROC for the central model. The central model, as well as the swarm and local models, were tested on the same test set with unseen data. We used a model trained on a single server with the whole dataset resulting from all the single datasets of nodes in the network. That is, when the new node joins the network, we evaluate the central model only once with 100% of the training set available. In the scenarios where the new node is not present, we calculated the central model over the portion of the dataset given to the nodes. That is, with 3 *other* nodes with a 20% data split for each, we calculated the central model over their overall 60% of the dataset. The central model performance is evaluated by averaging five repetitions with random shuffles of the training data with different random seeds, and we obtained an AUC of 0.8567 for *MIMIC-III*, and 0.8828 for *MIMIC-IV*.

#### AUC Comparisons

**Fig. 2:**
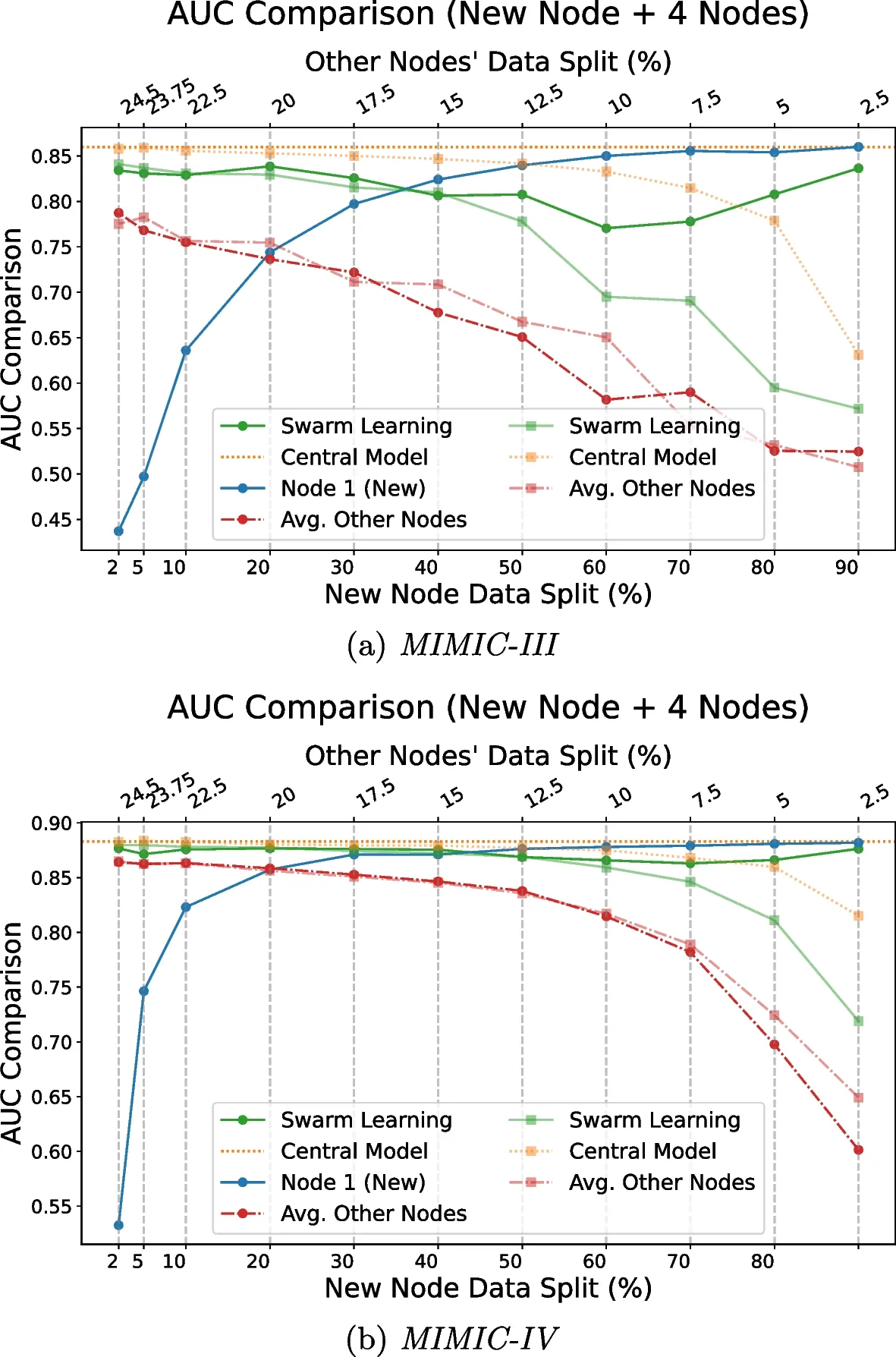
Avg. AUC in different data distribution scenarios. Here, it shows a scenario with 4 nodes (light colors and squared markers) and 4 nodes with the *new* node (solid colors and round markers), highlighting the performance of the new node (blue) against the other nodes (red), the SL (green), and the central model (orange)

The first swarm learning performance to show is the one obtained by the swarm network, the new nodes, and the other nodes before and after the new node joined and with different data distribution scenarios. Figure 2 represents one of these scenarios, and in particular the one with 4 nodes already part of the network and a new node, with a data distribution from 2.5% to 90% of the entire training set. With both *MIMIC-III* and *MIMIC-IV*, we can notice that the new node’s performance increased as its data distribution raised, with an AUC score very close to the central model with at least 70% of the data. On the other hand, the average performance of the other nodes decreased as we lowered their data distribution. Also, their score is very similar in the “before” and “after” comparisons, as their portion of data is unchanged, and the difference is due to the slightly different results obtained averaging the 5 random reshuffling. Across the five runs, the variability of the swarm AUC after the addition of the new node remained limited, with a standard deviation below 0.005 across all the considered configurations. Larger variability was observed only in the most data-limited local settings, particularly in *MIMIC-III*: when the new node contained 2.5% of the data, its local AUC was *0.638 ± 0.060* (mean ± SD) in the 2-to-3-node configuration and *0.665 ± 0.047* in the 4-to-5-node configuration. Similarly, when the four pre-existing nodes contained only 2.5% of the data each, the standard deviation of their average local AUC reached approximately 0.042.

Moreover, we could already observe that the lower number of admissions in this dataset negatively impacted the performance compared to *MIMIC-IV*. Indeed, this is also noticeable when we analyze the behavior for the swarm learning performance, where in *MIMIC-III* is generally lower than in *MIMIC-IV* across all the splits. However, here we may add two distinct observations. First of all, as for the impact of the *new* node on the swarm performance, it is clearly visible that, when the performances of the other nodes and their relative swarm performance rapidly decrease (the “before” scenario), the *new* node helps and, in the “after” scenario, the swarm performance always stays high and never fall. On the other side, although the swarm learning performance never reaches that of the central model, it is always superior to the performance obtained by the single nodes when they share a small or equal portion of data. For example, when all the nodes share the 20% of the dataset, the swarm learning performance is higher. We could also generally observe the same behavior in the “before” scenario, when all the *other* nodes always share the same amount of data.

**Fig. 3:**
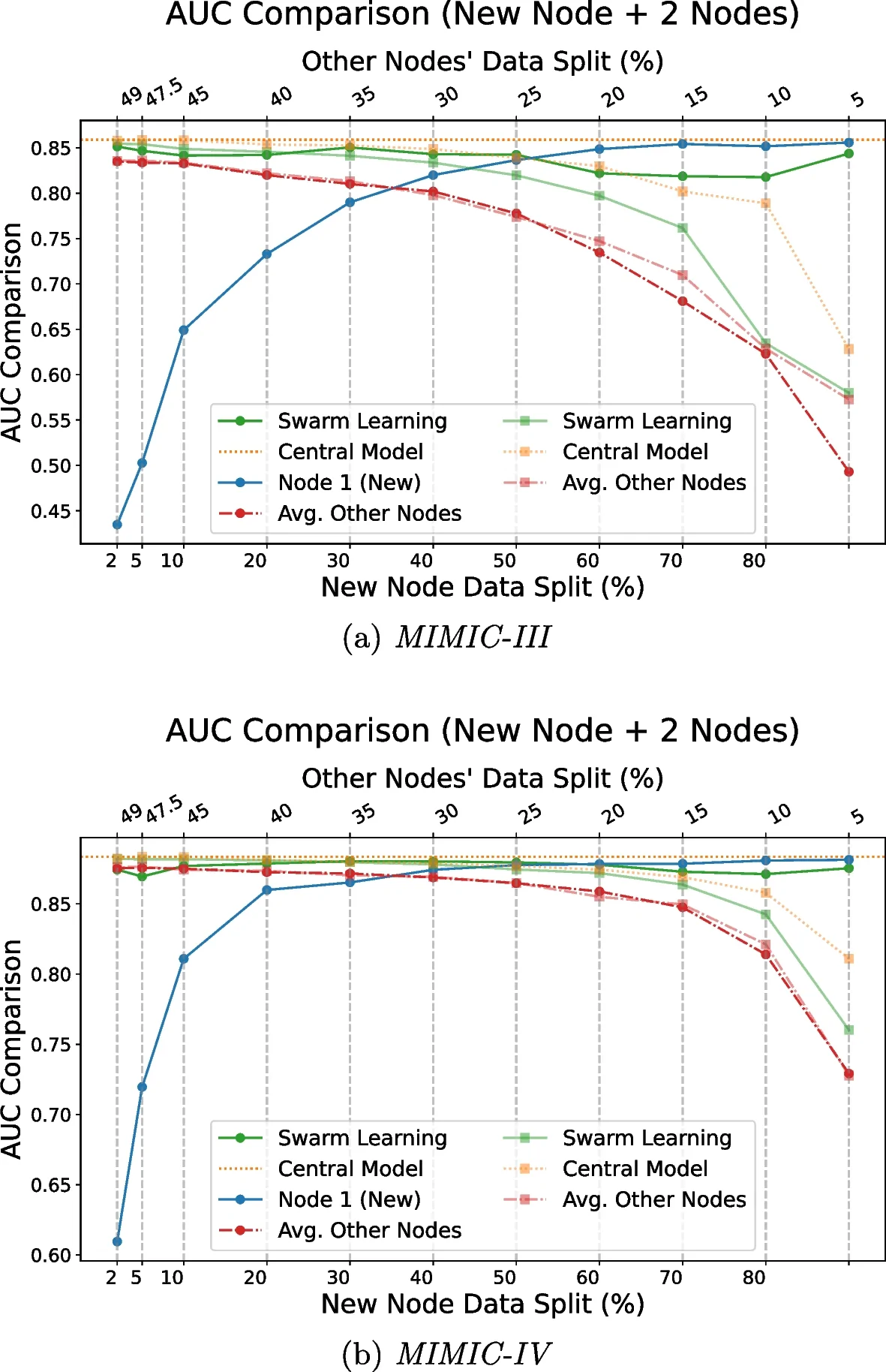
Swarm network average AUC performance comparison in a scenario with 2 nodes and 2 nodes with the *new* node

Figure 3 depicts an intermediate configuration with fewer nodes in the network, that is, 2 nodes and 2 nodes with the *new* node. The impact of the new node is always relevant, especially with a consistent volume of data (over 50% of the total) when it significantly improves the overall swarm learning performance both in the *MIMIC-III* and *MIMIC-IV* scenarios. Compared with the 4-node configuration, here we can observe that the swarm learning performance is almost identical, while there is an increase in the average performance of the other nodes, as in this configuration their data volume is doubled w.r.t. the scenario of Fig. 2. In *MIMIC-IV*, this advantage for the other nodes is reduced but still notable across all the splits, while in *MIMIC-III* the swarm learning performed on par with the average performance of the other nodes when their data volume is low (below 20% of the total dataset, which is 10% for each “other” node). This could suggest that, given a fixed volume of data to share across nodes, the swarm learning performance could benefit of many nodes.

#### Swarm Gain

**Fig. 4:**
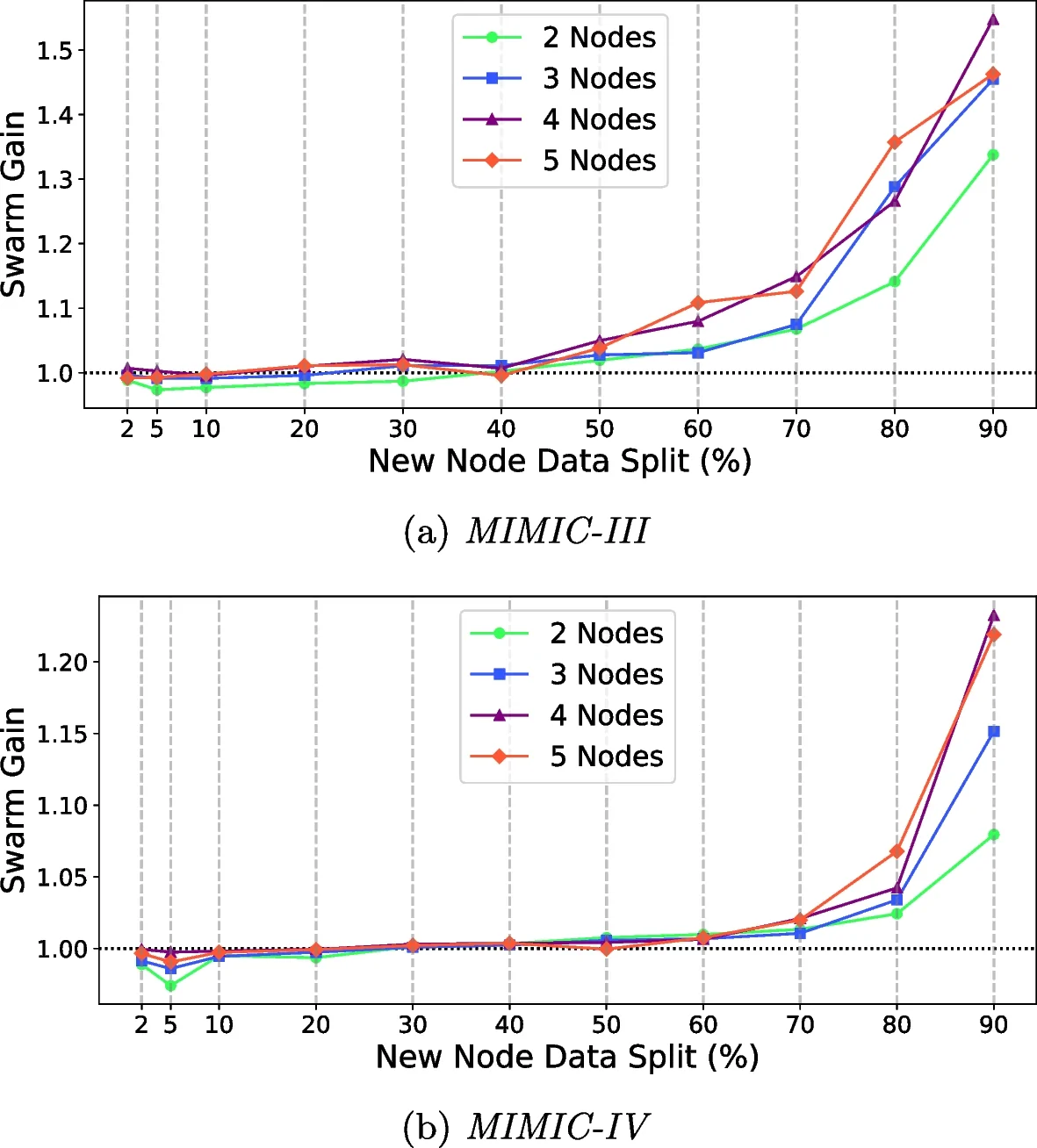
Swarm Gain score on *MIMIC-III* and *MIMIC-IV* data under different data distribution scenarios and with different node configurations

The swarm gain reflects an increasing swarm performance if the value is above 1. Figure 4 depicts the results on *MIMIC-III* and *MIMIC-IV* in a swarm network composed of 2 to 5 nodes. The first thing to notice is that the swarm gain value is never significantly lower than 1. That means that the swarm performance is never negatively influenced by the new node, regardless of the overall number of nodes in the network, even when the new node has a poorer performance (due to its small dataset) compared to the other nodes or the swarm itself. On the contrary, when the new node’s dataset is bigger than those of the other nodes, and its performance is higher, the swarm gain is up to 1.55 for *MIMIC-III* and 1.22 for *MIMIC-IV* (i.e., the swarm performance with the new node is up to 22% higher in *MIMIC-IV* and up to 55% higher in *MIMIC-III*).

Interestingly enough, the impact of the new node is greater in a configuration with more nodes. That is because the other nodes have to split equally the remaining part of the training set that is not used by the new node. More nodes correspond to a smaller data split, and the model is unable to perform well with few data. This is particularly noticeable in *MIMIC-III*, where the overall number of admissions considered is less than half of *MIMIC-IV*.

#### Local Gain

The local gain relates the local performance of the new node (i.e., the performance it can achieve on its own without joining the swarm network) to the performance of the swarm network with the node in it. Here, a value lower than 1 indicates a clear advantage for the node to join, whereas a value higher than 1 indicates that the node is performing better on its own.

**Fig. 5:**
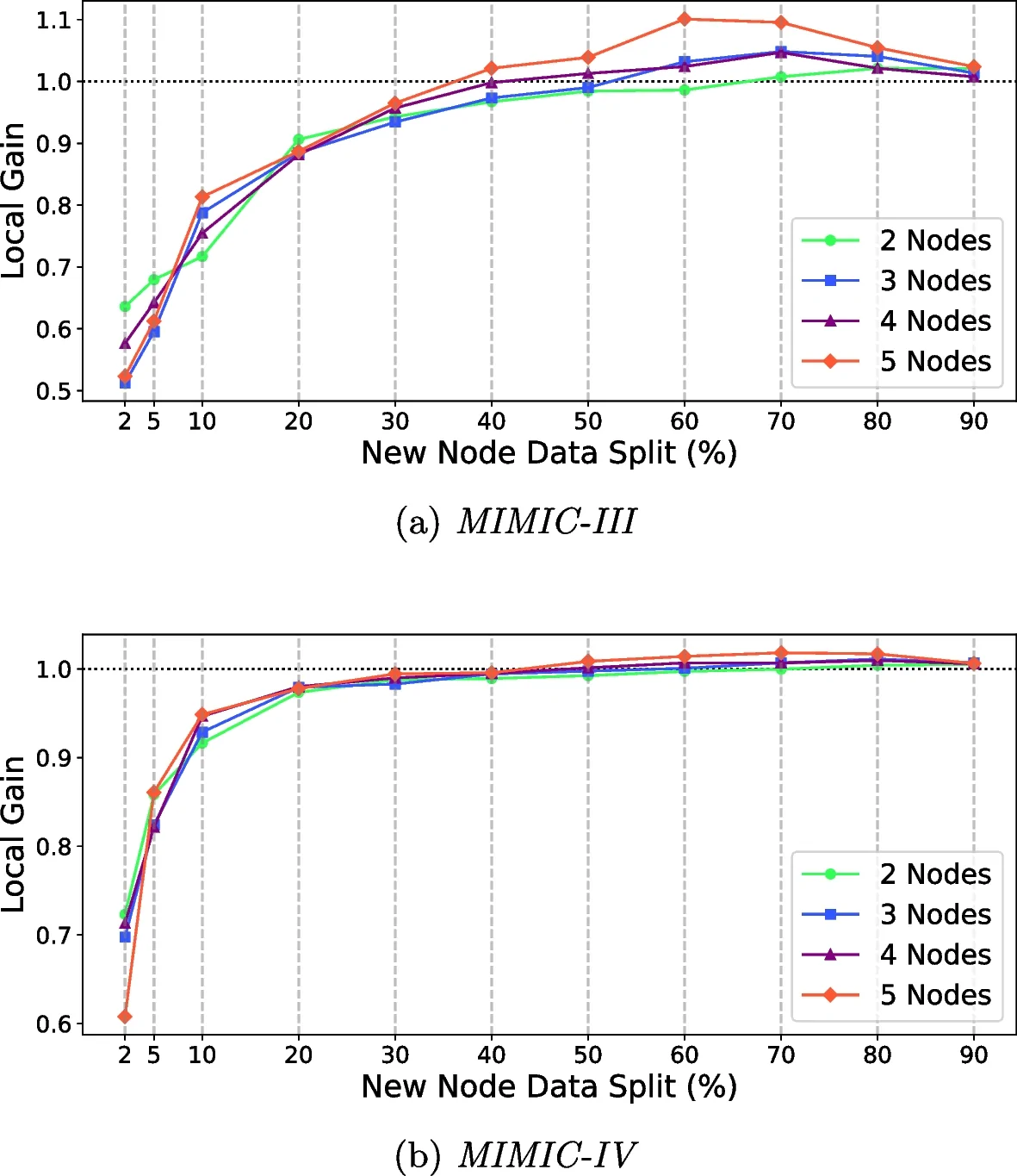
Local Gain score on *MIMIC-III* and *MIMIC-IV* data under different data distribution scenarios and with different node configurations

In Fig. 5, we can observe an interesting progress of the local gain based on the different data distribution scenarios. In fact, both in *MIMIC-III* and *MIMIC-IV*, it is beneficial for the new node to join the swarm network when its data is up to 30% of the total amount of data in the network, regardless of the number of nodes already in it. Beyond that threshold, the local gain stays close to 1, and it means that even with a larger data distribution, the new node does not suffer from a clear disadvantage from being in the network. This is particularly evident in *MIMIC-IV*. However, in *MIMIC-III* we can observe a peak around the 60-70% of data for the new node, especially in the configuration with more nodes $L_g \approx 1.1$, with values again around 1 over that amount of data. To explain this behavior, we have to analyze the individual performance of the nodes. In fact, the *new* node’s performances are almost identical for each data distribution considered after the 50%, as it reaches the limit given by the central model performance; while the other nodes’ performances are consistently decreasing as their datasets are diminishing in their dimensions. However, the swarm performance is calculated through a weighted average of the nodes’ performance with respect to their dataset dimensions, and the low weights of the other nodes do not provide a relevant loss in swarm performance. Thus, when the new node has 80%+ of the overall data, the impact of the other nodes is reduced.

#### Relative Local Gain

The relative local gain ($RL_g$) compares the swarm performance of the other nodes in the network before and after the new node joined, where a $RL_g > 0$ indicates an advantage for them to have the new node in the swarm network.

**Fig. 6:**
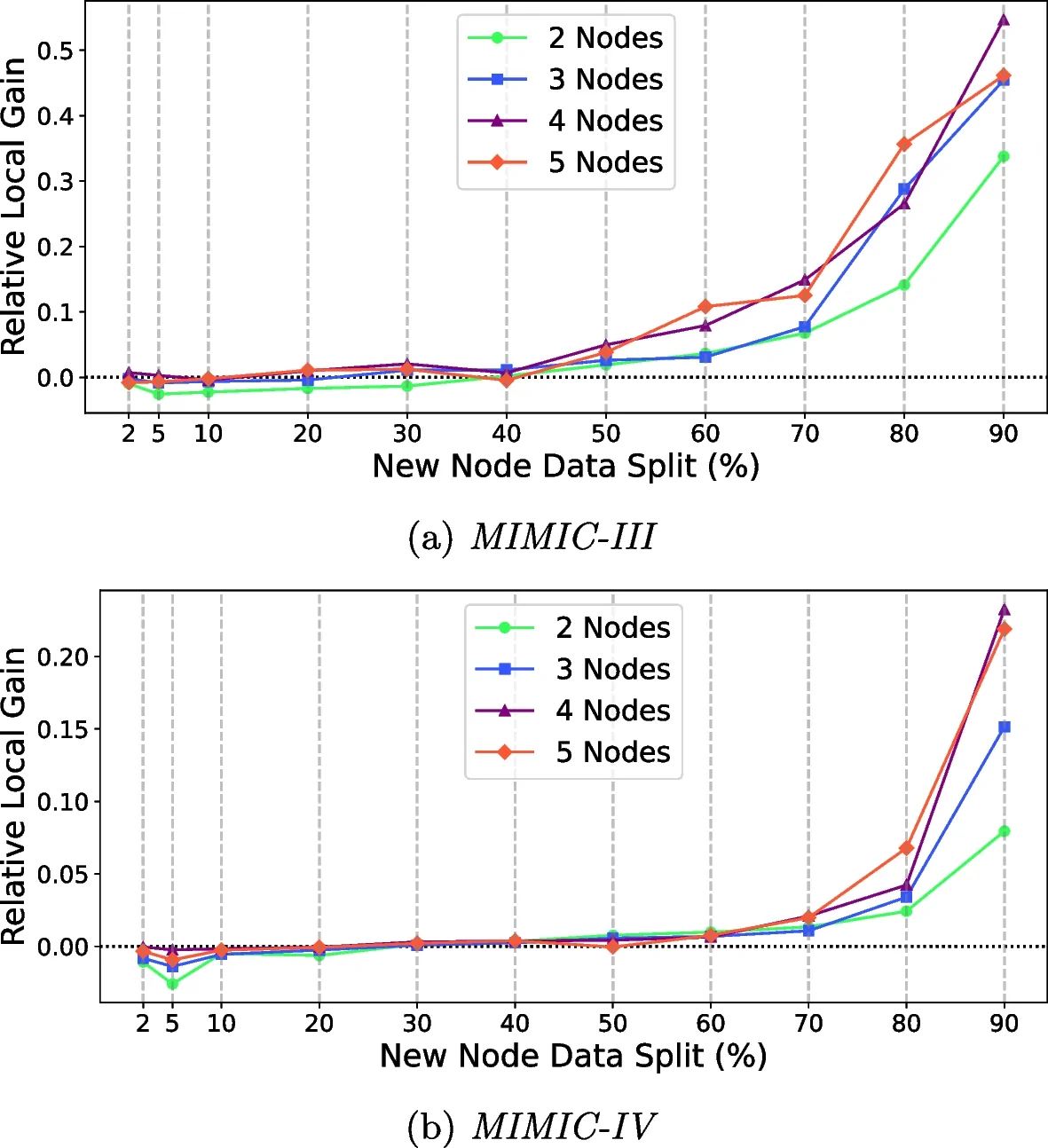
Relative Local Gain score on *MIMIC-III* and *MIMIC-IV* data under different data distribution scenarios and with different node configurations

As shown in Fig. 6, we never have a clear situation where it is not convenient for the other nodes to have a new node, regardless of the dataset dimensions considered or the overall number of nodes in the network. However, if the new node contributes with a particularly high portion of data (i.e., more than 50% of the total), it is particularly convenient for them, especially in a scenario with many nodes and *MIMIC-III*, where the other nodes’ swarm performance is up to 55% greater than before. Finally, as already discussed in Section 3.3, indicators $RL_g$ and $S_g$ could converge if the swarm performance is calculated through a weighted average and if the number of epochs adopted is sufficient. Because we are adopting the weighted average, we could infer that the number of epochs we are using, that is 25, is enough for both performances to converge, as the $RL_g$ shown in Fig. 6 is quite similar to the $S_g$ depicted in Fig. 4. Although, in the present setting, this similarity suggests that both metrics are capturing a similar global trend in swarm performance, the specific role of $RL_g$ and $S_g$ is different. $RL_g$ isolates the effect of adding the new node on the pre-existing nodes, which may become more informative in pre-convergence or asymmetric node-performance scenarios. Therefore, while the two KPIs partially overlap in the homogeneous converged regime considered here, they remain conceptually distinct indicators for more general swarm configurations.

#### Model Stability

The Model Stability Indicator (MSI) is used to verify if the new node introduces instability in the learning model, or if it stays consistent. A negative MSI signifies improved consistency in swarm learning, while a positive value indicates increased instability in model performance, potentially highlighting divergence caused by the new node. An MSI value close to zero implies minimal impact on model stability.

**Fig. 7:**
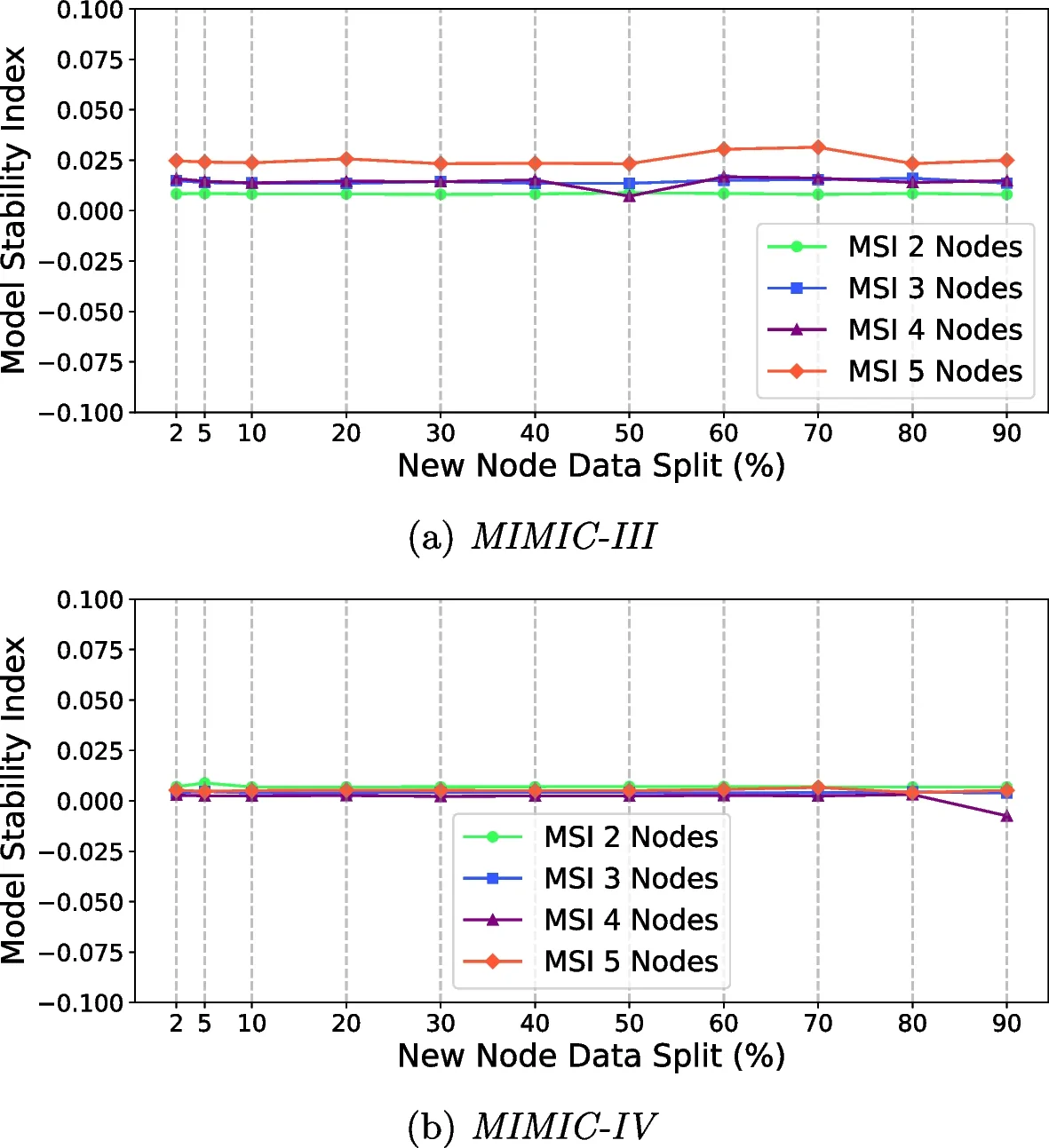
Model Stability Indicator on *MIMIC-III* and *MIMIC-IV* data under different data distribution scenarios and with different node configurations

As shown in Fig. 7, in our experiments, we have values very close to zero both for *MIMIC-III* and *MIMIC-IV*, regardless of the number of nodes considered. This can be explained by several factors: for example, in our experiments, we deliberately chose to use homogeneous data from *MIMIC-III* and *MIMIC-IV*. Consequently, the probability distributions that feed into the performance calculation will be nearly identical with the addition of one new node, as it does not introduce significant variability. Also, the ANN models used could be robust enough to overcome the changes in the training data given by the new node. Finally, in a swarm learning setting, the aggregation method should combine local updates from all nodes, thus reducing a possibly heavy influence of any one node. If, in our experiments, the collective performance remains stable when a new node is introduced, then we could suppose that the improvement in performance (if any) is due to the increased data volume rather than changes in performance variability. Accordingly, MSI should be interpreted within the fixed-budget training protocol adopted in this study, which was chosen to preserve comparability across nodes and configurations.

#### Jain’s Index

The Jain’s Index measures the fairness of the individual nodes in a distributed learning environment, and it is used to ensure that the distributed-global model is not heavily influenced by a single node. A value close to 1 denotes a performance contribution evenly distributed between nodes, while a lower Jain’s index implies a contribution imbalance, with a dominant node that is able to bias the aggregated model.

**Fig. 8:**
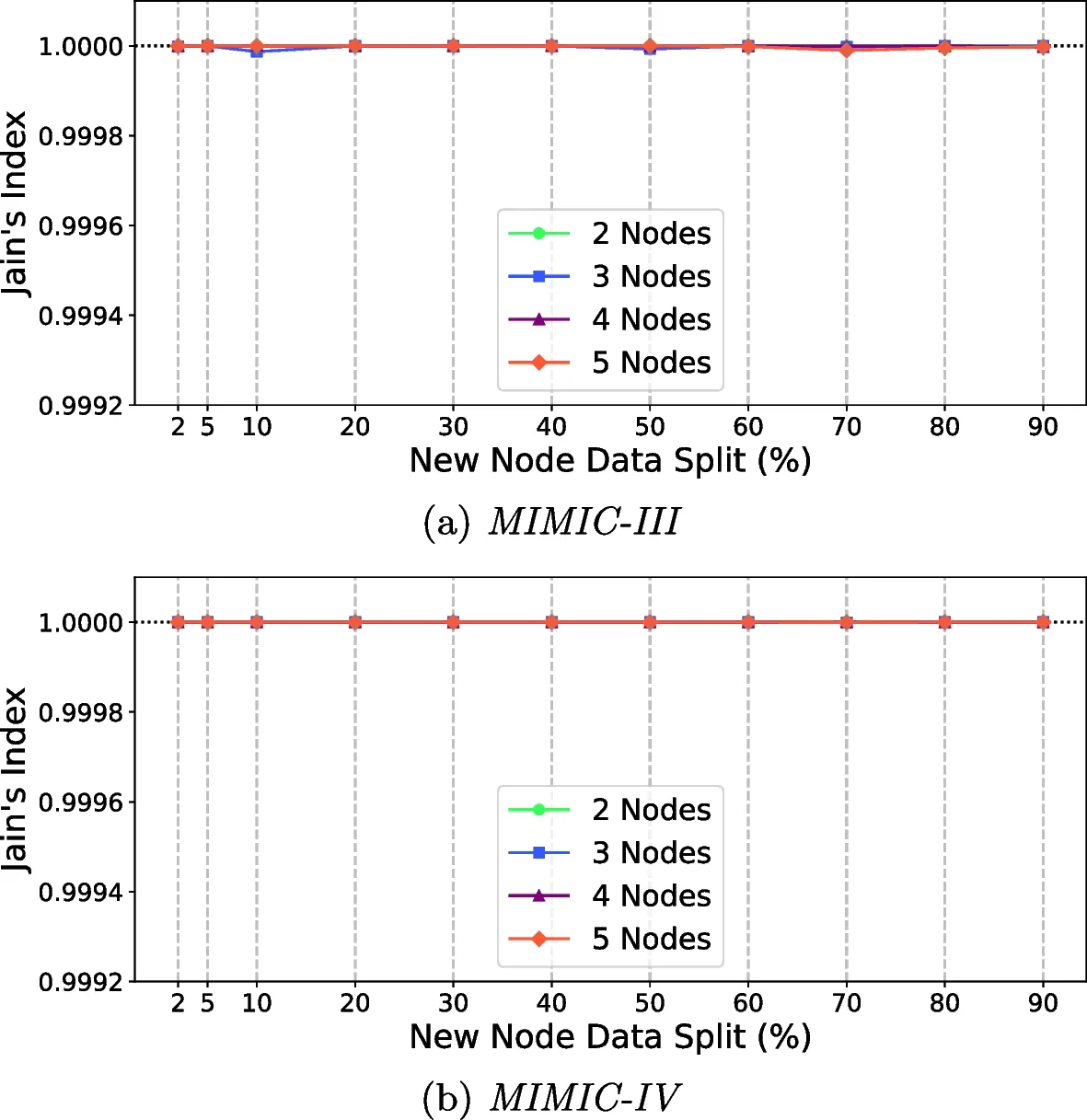
Jain’s Index on *MIMIC-III* and *MIMIC-IV* data under different data distribution scenarios and with different node configurations

Figure 8 shows Jain’s index for our experiments with homogeneous *MIMIC-III* and *MIMIC-IV* data, using different data splits for the new node and with different node configurations. It is immediately clear that, despite the new node contributing a variable fraction (from 2.5% to 90% of the overall dataset), the contribution from all nodes in the distributed learning system is almost perfectly balanced.

Moreover, as mentioned for the relative local gain, the training process proceeds over many epochs. Thus, the local models on each node tend to converge toward similar performance. This convergence through swarm aggregation further reinforces the fairness of contributions because any initial variability between nodes diminishes with time, leading to a robust and stable global model, but only if the number of epochs is adequate. Our experimental data shows that, regardless of the number of nodes considered, the homogeneous nature of the *MIMIC-III* and *MIMIC-IV* datasets combined with a distributed training over a proper number of epochs, results in a fair aggregated model. As a consequence, any improvement in the overall performance, such as an increased AUC, is more likely due to the benefit of additional data rather than to imbalances among the nodes. In fact, in the homogeneous setting adopted in this study, which serves as a controlled baseline, fairness and stability-related indicators are expected to show limited variation. This behavior should not be interpreted as a lack of usefulness of these KPIs, but rather as the expected reference behavior of the controlled baseline.

Since the additional node is designed not to introduce distributional heterogeneity, Jain’s Index and MSI characterize the fairness and stability profile of an idealized homogeneous node-addition scenario. Future non-IID evaluations can then be interpreted in relation to this baseline, with deviations from these values indicating the effect of feature shift, label shift, or institutional heterogeneity.

#### Data Utility Index

All the KPIs discussed above are showing the results of an ANN model trained through a swarm learning network in terms of performance and fairness, and from the point of view of a single node or on the whole swarm network. However, many indicators pointed out that the increase in performance is more related to a larger dataset than any other factor, and it is shown by the lower performances of *MIMIC-III*, especially with smaller nodes. Thus, it is fundamental to also analyze the data given to the swarm learning nodes. Both *MIMIC-III* and *MIMIC-IV* datasets are composed of 41 features and one target class, which is sepsis. As all the features are discretized, as described in Section 4.2, and the target class is stratified through all the nodes, it is almost certain that all the nodes contain all the possible labels for all the features. For this reason, we introduced the Data Utility Index, which considers the probability distribution of categorical features to evaluate if and how the new node’s label distribution differs from those of the existing nodes. A DUI close to 0 indicates identical distributions, whereas a higher value implies that the new node’s data distribution is significantly different from what is already present. Let us note that the DUI is an univariate index, thus it does not capture interactions between features or multivariate relationships among them, potentially missing how features jointly vary or correlate.

**Fig. 9:**
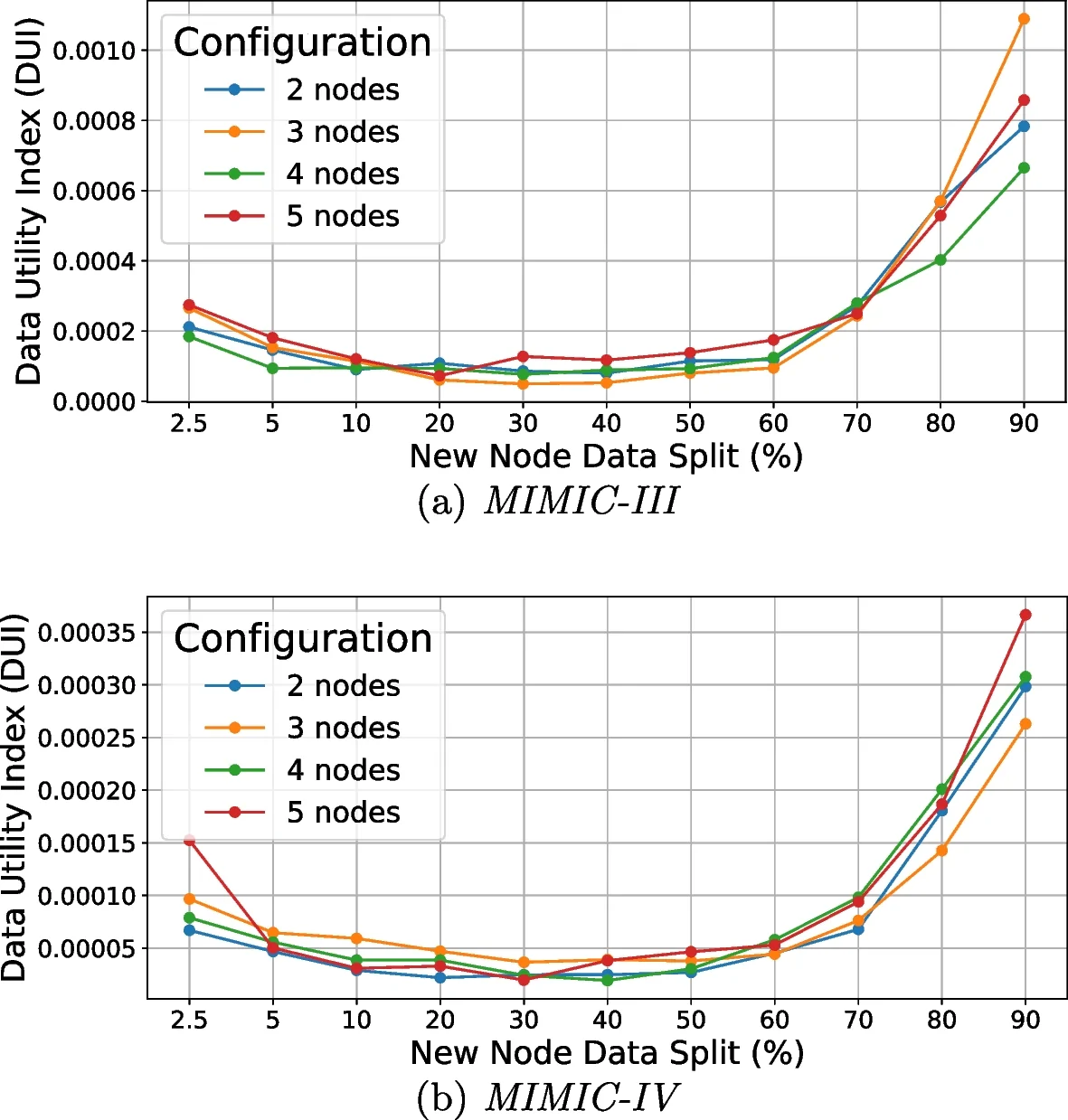
Data Utility Index score on *MIMIC-III* and *MIMIC-IV* data under different data distribution scenarios and with different node configurations

In our experimental results, shown in Fig. 9, we could observe that DUI is almost 0, indicating that the probability distributions of the labels in the new node are nearly identical to those of the other nodes. Since the DUI is calculated using a divergence metric—in our case, the Jensen-Shannon divergence between the new node’s label distribution and the aggregated distribution from the existing nodes, a value of 0 signifies no detectable divergence between them. This outcome highlights that in all the different data split scenarios considered, not only is the target class distribution preserved through stratification, but also all the other features’ distributions are maintained across the different datasets. Consequently, even if the new node contributes a variable fraction of the overall data (ranging from 2.5% to 90%), the underlying statistical properties across nodes remain consistent. These DUI values confirm once again that the new node does not introduce any novel information relative to the existing nodes, and therefore any improvements in model performance are more likely attributable to the increased volume of data.

#### Entropy

**Fig. 10:**
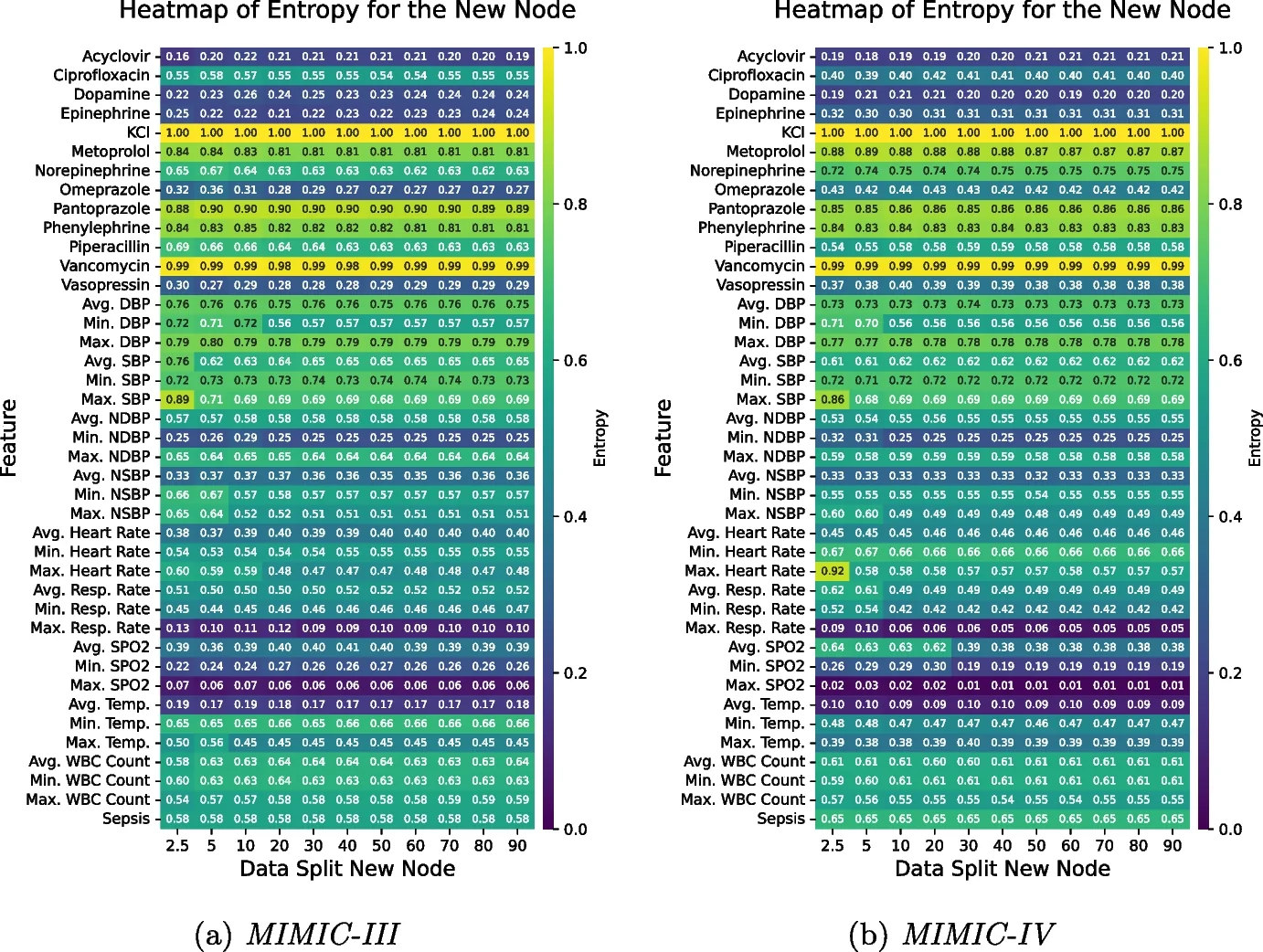
Entropy values for all the features on *MIMIC-III* and *MIMIC-IV* data under different data distribution scenarios

Entropy is another indicator used to analyze the input data for the *MIMIC-III* and *MIMIC-IV* datasets. Because our data are discretized, every node generally contains all possible values, such as “low,” “medium,” “high” for continuous variables that have been discretized (i.e., the vital signs) or “true”/“false” for binary indicators (i.e., the drugs considered). This ensures that the measure of entropy does not reflect the presence or absence of values, but rather the relative frequencies with which those values occur. When the entropy value is close to 1, we could infer that all the possible values for a certain feature are equally distributed; whereas with a value near 0, we could denote a feature that offers small variability and may be less discriminative when used for predictive modeling. Also, a feature with a very low entropy could not be very informative, as it contains almost only a few prevalent values, and the study of entropy is also potentially meaningful for feature selection. Since entropy depends on the selected features, their discretization, and the underlying dataset distribution, its values should not be interpreted as an absolute or dataset-independent measure of data quality. In the homogeneous scenarios considered in this study, the entropy analysis mainly serves to verify that the additional node is not associated with marked deviations in the discretized feature distribution. In fact, Fig. 10 depicts the entropy values for the *MIMIC-III* or *MIMIC-IV* data contained in the new node for all the features available, according to different data split scenarios, and with values considered in relation to the discretization thresholds adopted in the preprocessing workflow. First of all, we could observe that the entropy for all the features does not change significantly when the portion of data is big enough (e.g., at least 30% of the dataset in our case), and consequently, we could safely assume that the entropy shown for this new node over a certain threshold is the same entropy for the whole dataset. Then, we could start to observe some interesting issues. For example, KCl and Vancomycin have an entropy value close to 1 in both datasets, which suggests that these interventions are applied in a highly uniform manner on approximately 50% of the patients. In contrast, for medications such as acyclovir, dopamine, and epinephrine, a low entropy can reveal that nearly all patients receive (or do not receive) the treatment in a highly consistent manner. For what concerns the vital signs’ features, we could observe that the entropy for the blood pressure is high in both datasets, while the maximum SPO$_2$ has a very low entropy, which could reveal that nearly all patients achieve a normal oxygen saturation level (i.e., above 95%, according to Table 2) at least once during their stay, reflecting a desirable clinical outcome. Another interesting value to consider is related to the white blood cell count. We know that patients with sepsis have a significant variation in the WBC count (with an initial high count), and the entropy for WBC count and sepsis are very close in both *MIMIC-III* and *MIMIC-IV*, which could reflect a proper clinical sepsis diagnosis. On the other hand, it is more difficult to relate the sepsis entropy value with the ones of different medications, as some of them could be first-line medications while others may be used only as last-resort medications.

#### Comparison Between Different Neural Network Architectures

To assess whether the observed behavior of the proposed KPIs depends on the specific ANN architecture adopted in this study, we performed an additional set of experiments using the neural network architecture adopted in the original Swarm Learning study by Warnat-Herresthal et al. [7]. Their neural network consists of one input layer, eight hidden layers, and one output layer. The input layer is densely connected and is composed of 256 nodes, a ReLu activation function, and a dropout rate of 40%. From the first to the eighth hidden layer, nodes are reduced from 1,024 to 64 nodes, and all layers contain a ReLu activation function, a kernel regularization with an L2 regularization factor of 0.005, and a dropout rate of 30%. The output layer is densely connected and consists of one node and a sigmoid activation function. The model is configured for training with the Adam optimizer and for computing the binary cross-entropy loss between true and predicted labels.

**Fig. 11:**
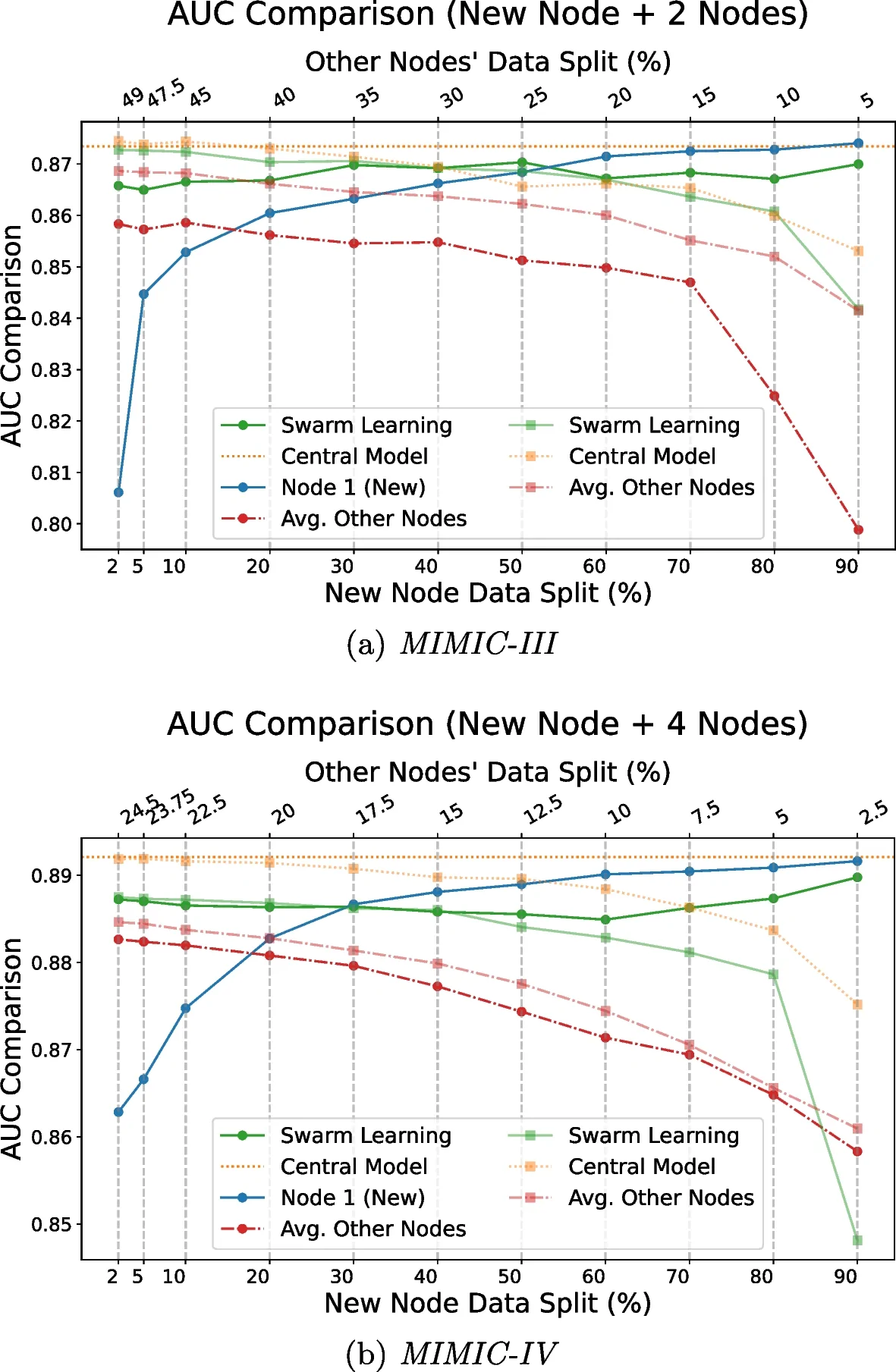
Swarm network average AUC performance adopting the neural network architecture used by Warnat-Herresthal et al. [7]

As shown in Fig. 11, the overall trends were consistent with those obtained with our baseline ANN. The absolute AUROC values were slightly higher, but the relative behavior remained substantially unchanged. The slight differences between the two “avg. other nodes” runs can be attributed to run-to-run variability caused by random initialization, batch ordering, and small differences in the swarm synchronization process, even when data, model architecture, activation functions, and aggregator are kept fixed. Although the behavior of the two different architectures is comparable, the execution time increased markedly, from approximately 110 hours to 469 hours, corresponding to an increase of about 326%. This highlights that the slight improvement in predictive performance is associated with a substantially higher computational cost, which is particularly relevant when considering the practical scalability of the evaluation. Since the present work focuses on the behavior of the proposed KPIs rather than on optimizing the predictive model architecture, we retained the simpler ANN as the main model in the manuscript.

## Conclusions

In this paper, we analyzed the impact of a *new* node on an already existing swarm learning network. To measure this impact, we proposed, as an original contribution, seven different key performance indicators (KPIs) designed to characterize the effect of the new node on different components of the system: the new node itself, the overall swarm network, and the pre-existing nodes, considering performance, stability, fairness, and data-related aspects. We deliberately adopted two homogeneous ICU datasets (*MIMIC-III* and *MIMIC-IV*) in order to evaluate the behavior of the proposed KPIs in a controlled setting, where the impact of the new node could be observed without the confounding influence of heterogeneity.

The results showed that the introduced KPIs are effective. Within this controlled setting, the proposed KPIs allowed us to characterize how the contribution of the new node changes according to the amount of data it provides. In the analyzed scenarios, we did not observe systematic performance degradation in the swarm network or in the pre-existing nodes after introducing the new node, across both datasets and data-volume configurations. Its contribution became particularly beneficial for the swarm when the amount of data provided by the new node exceeded a certain threshold, which was around 50% of the data in our experiments. Conversely, from the perspective of the new node itself, joining the swarm was most beneficial when its local data portion was relatively small compared with the total amount of data, approximately around 30% in our experiments. When the new node contributed the vast majority of the data, we observed cases in which its swarm performance was lower than the performance obtained using only its local data. However, in these same scenarios, the new node could still positively contribute to the pre-existing nodes, suggesting that the benefit of joining a swarm should not be assessed only from the perspective of the incoming node, but also in terms of its effect on the whole clinical network.

Overall, this study provides a controlled baseline for evaluating the impact of adding a new node to a swarm learning network. The homogeneous setting was not intended to reproduce the full complexity of real-world multi-center deployments, but to establish the expected behavior of the proposed KPIs when the new node does not introduce distributional heterogeneity. While the present validation focuses on homogeneous node-addition scenarios, the proposed KPI framework is intended to support the evaluation of broader swarm configurations. Future work will extend this analysis to heterogeneous scenarios, whose impact on performance and fairness has been highlighted in medical federated learning [22], in order to evaluate how the proposed indicators behave under more realistic conditions. In these settings, inter-node heterogeneity may alter the relationship observed here between data contribution and KPI behavior, with larger variations especially in stability-related indicators. As part of this investigation, it will be important to analyze cases in which the new node contains feature values or class distributions that are not represented in the pre-existing nodes. Further experiments could also assign data from different clinical centers to different nodes, allowing the adaptability of the swarm network to be assessed not only on real-world data, but also by simulating a real-world usage. Finally, since the objective of this work was to characterize the contribution of a new node within an SL configuration, future work could also address a direct comparison between SL and server-based federated learning approaches under matched aggregation and synchronization settings. Such a comparison would help clarify which observed effects are specifically related to the peer-to-peer coordination mechanism of SL and which are due to distributed parameter aggregation more generally.

## Data Availability

MIMIC III and MIMIC IV data are publicly available through the PhysioNet portal.

## Footnotes

1 https://github.com/HewlettPackard/swarm-learning

2 https://github.com/HewlettPackard/swarm-learning/blob/master/docs/User/How_to_Swarm_enable_an_ML_algorithm.md
